# The Impact of the Azo-Chromophore Sort on the Features of the Supramolecular Azopolyimide Films Desired to Be Used as Substrates for Flexible Electronics

**DOI:** 10.3390/ijms232315223

**Published:** 2022-12-03

**Authors:** Iuliana Stoica, Elena-Luiza Epure, Andreea Irina Barzic, Ilarion Mihaila, Catalin-Paul Constantin, Ion Sava

**Affiliations:** 1 Department of Physical Chemistry of Polymers, “Petru Poni” Institute of Macromolecular Chemistry, 700487 Iasi, Romania; 2 Faculty of Chemical Engineering & Environmental Protection, “Gheorghe Asachi” Technical University, 700050 Iasi, Romania; 3 Integrated Center of Environmental Science Studies in the North-Eastern Development Region (CERNESIM), “Alexandru Ioan Cuza” University of Iasi, 700506 Iasi, Romania; 4 Department of Electroactive Polymers and Plasmochemistry, “Petru Poni” Institute of Macromolecular Chemistry, 700487 Iasi, Romania

**Keywords:** azo-chromophore, supramolecular system, flexible azopolyimide film, colorimetry, surface relief grating, atomic force microscopy, anisotropy, molecular modeling

## Abstract

High-performance supramolecular polyimide systems were synthesized via a simple and innovative approach using two types of azo-chromophores, leading to concomitant special properties: high thermostability, the ability to be processed in the form of films with high flexibility, adequate morphological features, and good structuring capacity via phase mask ultraviolet (UV) laser irradiation, induced by the presence of the azo groups (–N=N–). The dimension and the anisotropy degree of the micro/nano patterns obtained on the surface of the flexible films (determined by atomic force microscopy) depend on the azo-dye type used in the supramolecular azopolyimide synthesis, which were higher when the azo-chromophore containing a –cyano group (–C≡N) was used. The molecular dynamics method, an excellent tool for an in-depth examination of the intermolecular interactions, was used to explain the morphological aspects. Energetic, dynamic and structural parameters were calculated for the two systems containing azo-chromophores, as well as for the pristine polymer system. It was highlighted that the van der Waals forces make a major contribution to the intermolecular interactions. The results from the combination of the dynamic analysis and the concentration profile explain the better mobility of the polyimide chains with a maximum content of azo groups in the *cis* configuration compared to the other systems. Taking all these data into account, the surfaces of the films can be tuned as required for the proposed applications, namely as substrates for flexible electronis.

## 1. Introduction

The advances of many actual technologies are dictated by the breakthroughs made on each type of material composing the product. Polymers are largely employed in the fabrication of numerous devices, which are indispensable in our daily life [1,2]. The achievement of novel materials with updated performance requires deep fundamental research at the molecular level to understand the factors and processes that are capable of contributing to the control and/or adaptation of the final properties [3,4]. A particular interest is attributed to polymers that contain photodeformable sequences or additives, which enable the achieving of specific changes at the molecular level via radiation exposure [5,6,7]. Azo-derivatives are often linked or introduced in a polymer host to render photo-responsive characteristics [8]. In precise irradiation conditions, such substances can pass from one isomeric state to another (*trans*-*cis* or reverse). Polarized radiations with wavelengths within UV or VIS ranges generate uniaxial orientation of the azo-based molecule: multiple photo-induced isomerizations between the two known configurations of the azo moiety determine a change in the position of the transition dipole of the chromophore [9]. Hence, the polymer systems gain birefringence, having the radiation-induced optical axis with a distinct orientation in regard to the polarization sense of the actinic radiation [9]. In other words, polarized light interaction with the azo-polymers renders dichroism, birefringence, significant changes in morphology (known as surface relief gratings (SRGs)), and photomechanical outcome (in free-standing polymer films) [10,11]. SRGs’ fabrication on azo-polymers is a phenomenon largely examined and it is affected by the conditions of production of the periodic optical pattern (wavelength, fluency, and type of radiation polarization). In any case, the mechanisms underlying SRG formation are partially identified, but the contributing physical processes are not deeply clarified. Some reports state that in high glass transition polymers, the SRGs are the outcome of optically induced mass transport with insignificant thermal effects [12], whereas others take into account thermal ablation and photodegradation processes [13]. Production of bulk birefringence and engraving of SRGs in azo-polymer systems afford their use in holographic devices [14], liquid crystal displays [15], optical switching [16], light couplers for waveguides [17], optical storage [18], and shape memory devices [19]. Based on the above described molecular phenomena, the researchers were focused on the development of SRGs on several kinds of polymer structures containing azo-chromophores, such as modified chitosan [20], polymer salt [21], polymethyl methacrylate [22], polystyrene [23], poly(vinyl alcohol) with azobenzene side chains [24], and polyimides (PIs) [25]. The latter category of polymers introduces the advantage of high thermal and radiation stability, while they exhibit attractive mechanical and electrical performances [26,27]. Related to the topic of SRG creation on PI layers, one of the incipient studies was made in 1999 by Chen et al. [28], which employed an azocarbazole-based polyimide on which they made SRGs. The presence of dopants enhanced the absorbance at 488 nm. Depending on the light polarization state, they can generate or erase the birefringence. The formation of SRGs under circularly polarized laser treatment proved to be highly thermally stable without additional changes in surface features while baking over 200 °C. Another relevant study on SRGs formed on polyimides was performed by Li et al. [29]. They emphasized the role of laser UV irradiation history on the polyimide surface topography. For PI synthesis, a photo-sensitive dianhydride was used. First, they irradiated the sample at a fluence below the threshold and then the periodic surface structure was generated in two stages, one involving small fluence (under the threshold) and the second was performed at variable fluences that denote the limits of no SRG formation and their deterioration. However, in this work, Li et al. [29] did not include an azo-derivative. On the other hand, the creation of SRG PIs with nano-features is considered an almost impossible goal to reach. For this purpose, a synergism among concepts of chemical design, surface engineering, and laser physics must be simultaneously considered to attain a supramolecular polymer architecture that renders to the desired PI film morphology. The strategy of SRG preparation on azo-PI layers via the hydrogen-bonded supramolecular arrangement of PI chains had been first evidenced by the group of E. Schab-Balcerzak [30,31,32,33,34,35]. They employed chromophores which included a pyridine ring as a replacement for the phenyl unit of azobenzene or a matrix modified to comprise pyridine. Their investigations underlined the photoresponsive characteristics of azopyridine-derived PIs, but they analyzed particular excitation conditions and only a few imide polymer structures. As far as we know, the influence of the azo-derivative structure on tuning the resulted PI supramolecular architecture was not discussed yet in the literature. 

This work attempts to explore new aspects derived from the features of the supramolecular azopolyimides, emphasizing the influence of the azo-monomers type, namely 4-[(4-methylphenyl)diazenyl]phenol (AzoCH_3_) or 4-[(4-cyanophenyl)diazenyl]phenol (AzoCN) that were mixed with the polyamidic acid (PAA). Special attention is paid to the selection of the proper components that will lead to the supramolecular azopolyimides, since the goal was to obtain a concomitant good piezo response (induced by the –CN group) and good behavior in the formation of high SRGs (induced by the azo compound). The formation of the intermolecular interactions between azomonomer and polymer host allows the increase of the chromophore concentration over the solubility limit of the doped systems, and for preventing disadvantageous chromophore evaporation or phase separation. The obtained supramolecular systems in the form of thermostable flexible films are optically, thermally, and morphologically tested to elucidate their applicability as interfaces in flexible electronics. A series of simplified models are built for the purpose of characterizing the polymer systems by the molecular modeling method. Different energetic, dynamic, and structural parameters are used to understand the behavior of the investigated polymers at the nanoscale level during the formation of morphological patterns derived from the structuring capacity via phase mask UV laser irradiation.

## 2. Results and Discussion

**Figure 1:**
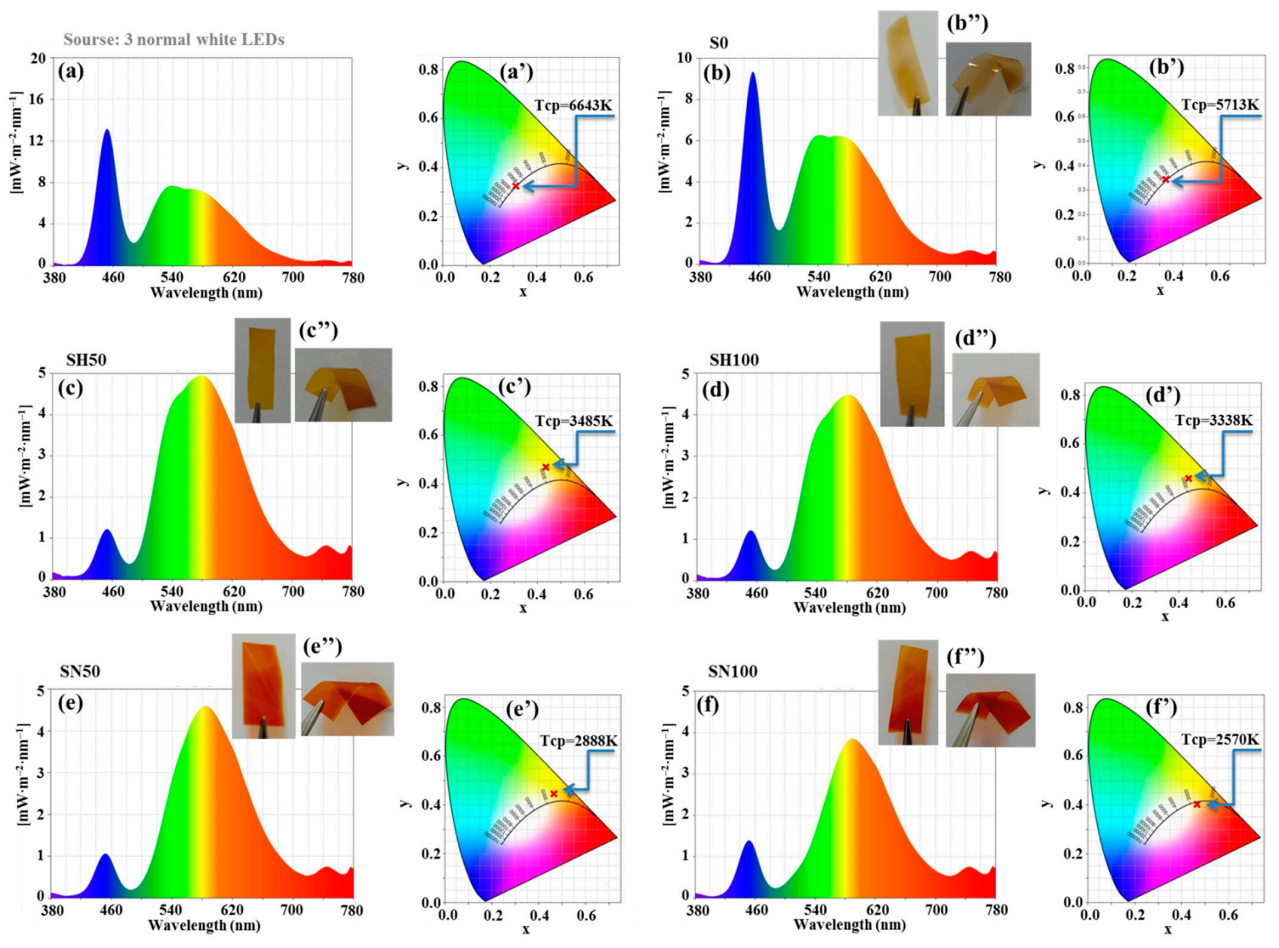
Spectral distributions in the visible light range and CIE 1931 xy chromaticity diagrams registered without sample, using three normal light LEDs (**a**,**a’**) and for samples S0 (**b**,**b’**), SH50 (**c**,**c’**), SH100 (**d**,**d’**), SN50 (**e**,**e’**), SN100 (**f**,**f’**). The inserted pictures present each flexible film before and after bending/torsion (**b’’**—S0, **c’’**—SH50, **d’’**—SH100, **e’’**—SN50, **f’’**—SN100).

The polyimide films obtained by the proposed synthesis method, with a thickness of about 30 ± 5 µm, were all very flexible, resisting at combined bending and torsion tests, and returning easily to their initial state. In Figure 1, the aspect of each flexible film can be observed, both in initial and in bending/distorted states. The films showed good resistance to the solvents used in the cleaning process to which the flexible substrates are subjected before fabricating various electronic devices over them. 

### 2.1. Colorimetry Study

**Figure A1:**
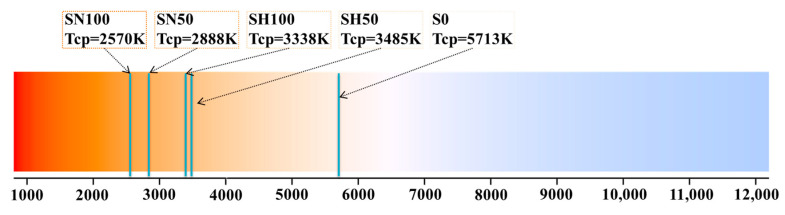
The place of the pristine polyimide and supramolecular azo-polyimides films on the color temperature scale.

Another observation regarding the appearance of the polymer films is the color influenced by the type and the quantity of azo-chromophore used in the synthesis. This colorimetry aspect is revealed by the spectral distributions in the visible light range (Figure 1a–f) and CIE 1931 xy chromaticity diagrams (Figure 1a’–f’). These data were registered first without any sample, using three normal light LEDs, and then with each sample: pristine polyimide (S0), supramolecular systems with AzoCH_3_ (SH), and supramolecular systems with AzoCN (SN). The molar ratio between the polyamidic acid and the azomonomer was 1:0.5 (SH50, SN50) and 1:1 (SH100; SN100). According to the values of the peak wavelength (used as an indicator of the samples hue) determined from spectral distributions in the visible light range and displayed in Table 1, the supramolecular materials with AzoCH_3_ have a shade of yellow, while supramolecular materials with AzoCN are orange in hue. The coloring was more intense as the azo-dye concentration increased. The color temperature registered for the pristine polyimide film, determined from the CIE 1931 chromaticity diagrams in xy coordinates, was higher when compared with those obtained for the azo-polyimide films (and Table 1), indicating that the sample is very transparent. Figure A1 from Appendix A showcases the place of the pristine polyimide and each supramolecular azo-polyimide film on the color temperature scale. The data are in accordance with the ones resulting from spectral distributions. In addition, as the color of the samples became slightly darker in yellow or orange tone, the quantity of light passing through the sample is lower, with the illuminance being 278 lx for SH50 and only 170 lx for SN100. The ability of the films of azo-polyimides with supramolecular organization to transmit electromagnetic energy (the transmittance) was influenced by the measured illuminance, varying, according to Table 1, from 83% for S0 to 52 and 36% for the samples with a large quantity of AzoCH_3_ and AzoCN, respectively. 

### 2.2. Thermal Stability

**Figure 2:**
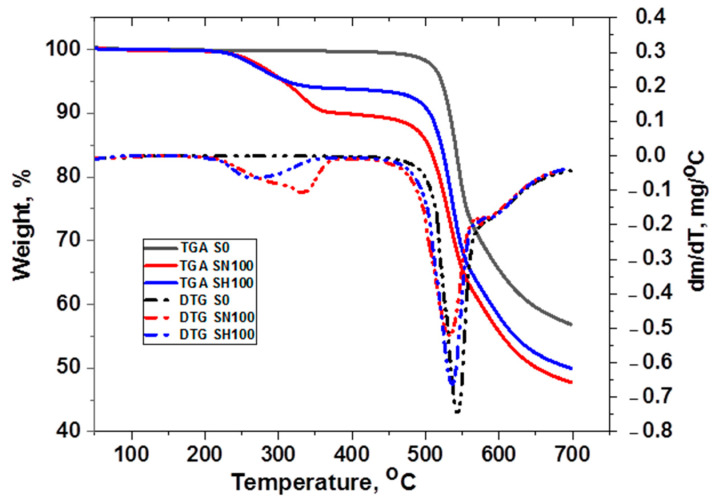
TGA and DTG of pristine polyimide S0 and of supramolecular structures of SN100 and SH100.

The thermal stability of the pristine polyimide S0 and supramolecular structures SH100 and SN100 was evaluated by thermogravimetric analysis (Figure 2, Table 2). All the polymers under investigation revealed great thermal stability. The thermal degradation onset temperature (T_onset_) decreases from 512 °C for pristine polyimide S0 to lower values, but still above 225 °C when adding different types of azo-chromophore in the supramolecular structures. The polymer containing AzoCN presents a higher T_onset_ compared to the polymer containing AzoCH_3_. The presence of the azo groups determines the first step of degradation at a temperature (T_5_) of 308 °C, which is unobservable for the pristine polyimide. The second step starts at about 500–510 °C and is characterized by DTG peak at about 530 °C. This step represents the main decomposition process. This last step can be attributed to the degradation of macromolecular chains of the polymer and take place at high temperatures.

### 2.3. Morphological Study of UV-Laser Induced Modulations

**Figure A2:**
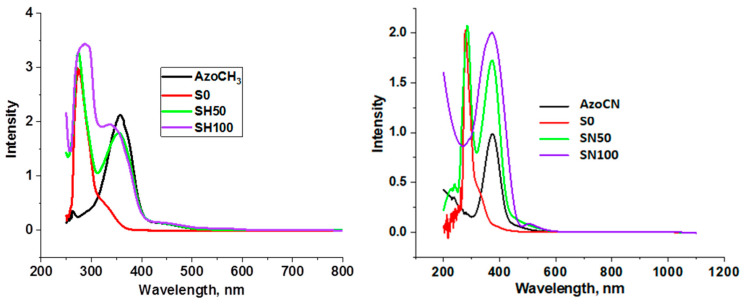
UV-Vis absorption spectra of the supramolecular azo-polyimide SH and SN vs AzoCH_3_, AzoCN and pristine polyimide S0.

**Figure A3:**
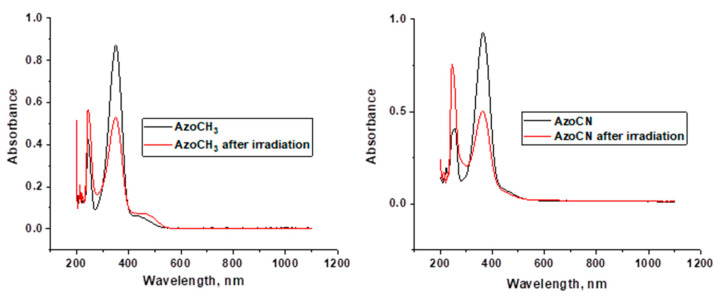
UV-Vis spectra of AzoCH_3_ and AzoCN.

**Figure A4:**
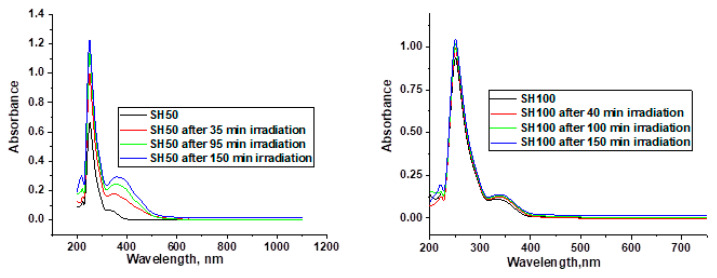
UV-Vis spectra of SH50 and SH100.

**Figure A5:**
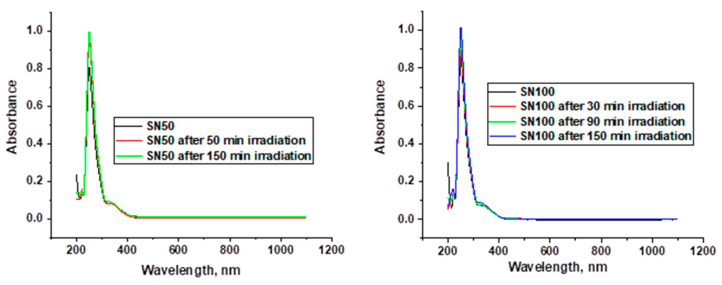
UV-Vis spectra of SN50 and SN100.

The supramolecular azo-polyimide systems are desired to be tested in the future as substrates for applications in flexible polymer memory devices. In this context, it is necessary to improve the surface of the flexible films by inducing a micro pattern on a generous area in order to enhance the data storage ability. For this purpose, a further goal was to check the capability of the azo-polyimide films to form surface relief gratings on the action of the UV laser, through a phase mask with grooves. To identify the laser working wavelength that will give the best results, UV-Vis spectroscopy was performed (data presented in Appendix B, Figure A2, Figure A3, Figure A4 and Figure A5). The UV-Vis absorption spectra of the two types of supramolecular azo-polyimides SH and SN presented peaks found in the wavelength range of 340–360 nm. These were induced by the presence of each azo-dye AzoCH_3_ and AzoCN (associated with the *trans* isomer symmetry-allowed π–π* transition), and compared to the pristine sample S0 where no modifications were observed in this area. The intensity of each band increased as the molar ratio between the polyamidic acid and the azomonomer increased from 1:0.5 (SH50 and SN50) to 1:1 (SH100 and SN100). This was an indicator that the proper working wavelength for the patterning experiments will be the third harmonic of a pulsed Nd:YAG laser (355 nm). In addition, from our previous experience [15,36,37,38], we could choose the most suitable irradiation conditions to study the influence of the azo-chromophore type on the resulting micro/nano structures (presented in the Experimental part). 

**Figure 3:**
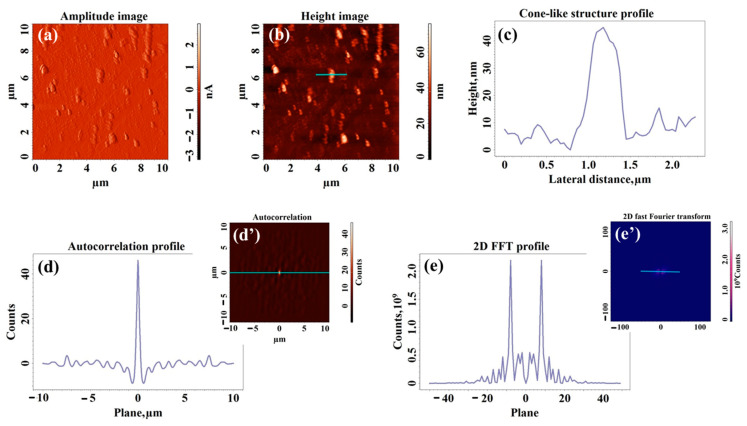
AFM morphological analysis of laser-irradiated pristine polyimide S0: (**a**) amplitude image, (**b**) topographical image, (**c**) cross-section profile taken along the line from height image, (**d**) autocorrelation profile collected along the profile line from autocorrelation image (**d’**), (**e**) two-dimensional fast Fourier transform profile collected along the profile line from two-dimensional fast Fourier transform image (**e’**).

The morphological aspects of the surface modifications observed after phase mask UV laser irradiation were investigated using atomic force microscopy. Figure 3 shows 10 × 10 μm^2^ AFM amplitude (a) and topography (b) image of the laser-irradiated pristine polyimide S0. The semicontact error mode that gives the amplitude image was used to better observe the fine details of the resulting morphology that can be omitted only when the height image is analyzed. Since S0 does not contain an azo-component in its chemical composition, well-developed cone-shaped structures were found on the investigated surface. These formations of around 45 nm in height and 670 nm in width (values calculated from multiple cross-section profiles like the one from Figure 3c) were sparsely distributed, but in some regions were densely packed. Similar formations on polyimide films induced by the laser were found also in the literature [39,40,41], with the irradiation conditions influencing their dimensions and aspect. The occurrence of these cone-like arrangements is induced by the self-organization of polyimide molecules and clusters under UV laser irradiation. Due to their small heights and reduced complexity, the values of the surface roughness (Sq) and surface area ratio (Sdr) [42] remained low (Table 3). The random disposal of the cone-like morphology features induced on the modified surface an overall mild isotropic character, highlighted by the value of the texture direction index (Stdi) as presented in Table 3. In addition, this moderate isotropic nature was shown by analyzing the autocorrelation profile (Figure 3d) collected along the profile line from the autocorrelation image (Figure 3d’). This was obtained using Image Analysis software starting from the fast Fourier transformation (FFT) of the source data Z(X,Y)—see the two-dimensional fast Fourier transform profile (Figure 3e) and the two-dimensional fast Fourier transform image (Figure 3e’). The random aspect of these representations together with the high value of the texture aspect ratio parameter (Str) [43] indicates the unevenness of the orientation of the morphological structures resulted for S0 after the laser irradiation. 

**Figure 4:**
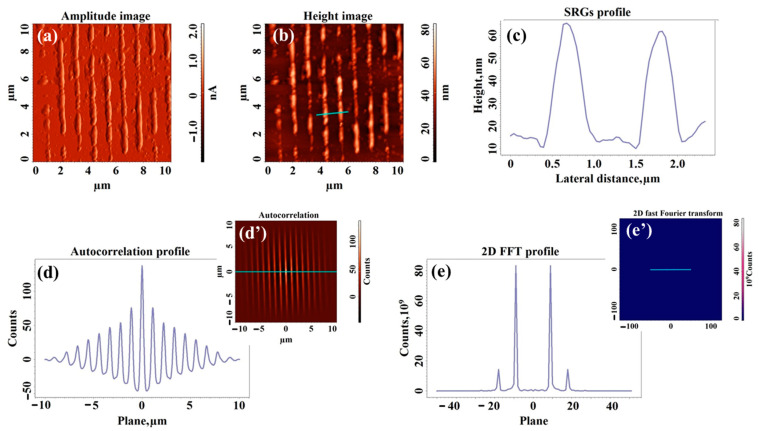
AFM morphological analysis of laser-irradiated SH50 supramolecular azo-polyimide films: (**a**) amplitude image, (**b**) topographical image, (**c**) cross-section profile taken along the line from height image, (**d**) autocorrelation profile collected along the profile line from autocorrelation image (**d’**), (**e**) two-dimensional fast Fourier transform profile collected along the profile line from two-dimensional fast Fourier transform image (**e’**).

**Figure 5:**
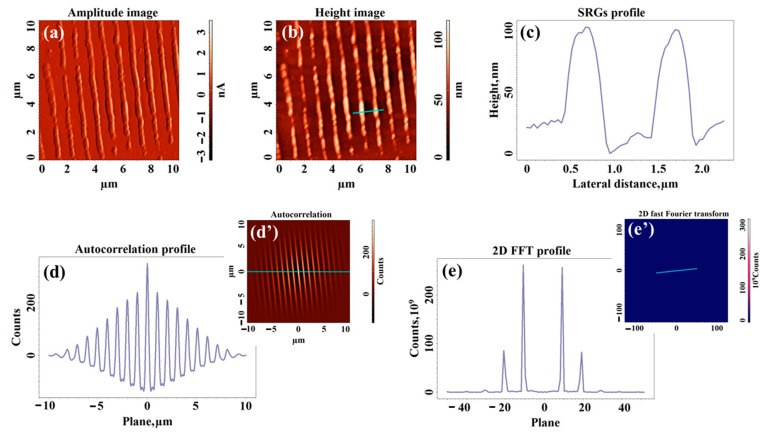
AFM morphological analysis of laser-irradiated SH100 supramolecular azo-polyimide films: (**a**) amplitude image, (**b**) topographical image, (**c**) cross-section profile taken along the line from height image, (**d**) autocorrelation profile collected along the profile line from autocorrelation image (**d’**), (**e**) two-dimensional fast Fourier transform profile collected along the profile line from two-dimensional fast Fourier transform image (**e’**).

**Figure 6:**
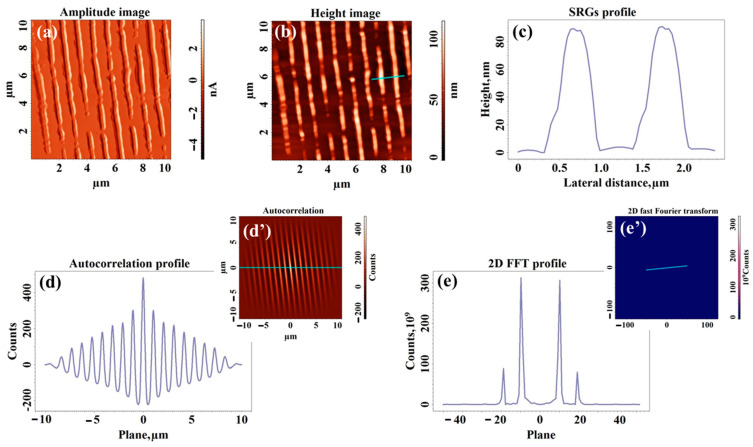
AFM morphological analysis of laser-irradiated SN50 supramolecular azo-polyimide films: (**a**) amplitude image, (**b**) topographical image, (**c**) cross-section profile taken along the line from height image, (**d**) autocorrelation profile collected along the profile line from autocorrelation image (**d’**), (**e**) two-dimensional fast Fourier transform profile collected along the profile line from two-dimensional fast Fourier transform image (**e’**).

**Figure 7:**
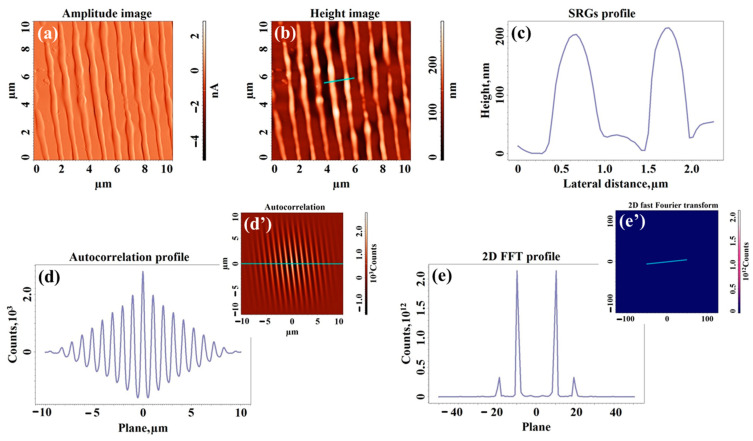
AFM morphological analysis of laser-irradiated SN100 supramolecular azo-polyimide films: (**a**) amplitude image, (**b**) topographical image, (**c**) cross-section profile taken along the line from height image, (**d**) autocorrelation profile collected along the profile line from autocorrelation image (**d’**), (**e**) two-dimensional fast Fourier transform profile collected along the profile line from two-dimensional fast Fourier transform image (**e’**).

Instead, when the supramolecular azo-polyimide films were irradiated using a linear diffraction grating with 1000 lines/mm, surface relief gratings started to form (Figure 4, Figure 5, Figure 6 and Figure 7). The periodicity of the pattern of around 1 μm was imposed by the phase mask characteristics. According to the specialized literature, there are several reasonable nanostructuration mechanisms that can act in the formation of these patterns: namely, an extremely quick process of supramolecular reorganization induced by the orientation of the azo-groups dipoles [44], a material photo-fluidization caused in the exposed regions by multiple *trans*-*cis* isomerization processes of the azo-segments [45,46], a dislocation of the matter from exposed to unexposed regions of the linear diffraction grating [45], and an opposite mass movement from unexposed to exposed areas [45]. These mechanisms can synergistically act, but the contribution of each of them can be in a different proportion. In particular cases, a certain process can be prevalent depending on the structure of the polymer and the irradiation conditions, leading to surface relief gratings with special structuring characteristics. Thus, the morphological aspect of the relief can sometimes explain which mechanism is dominant. Analyzing Figure 4 and Figure 5, where the results for the SH-type supramolecular azo-polyimide are displayed, it can be observed that when the molar ratio between the polyamidic acid and azomonomer was 1:0.5, the SRGs show discontinuities in some regions and a spiral-like aspect (Figure 4a,b). Their average heights and widths are around 50 nm and 590 nm, calculated from the cross-section profiles (see Figure 4c and Table 3), respectively. In addition, between the modulations, traces of unorganized material can be noted, especially in the amplitude images. Instead, when the molar ratio was 1:1, the higher quantity of azo-chromophore makes SH100 react better during UV-laser irradiation. SRGs were better defined, slightly narrower, and presenting fewer interruptions and heights of almost double value (Figure 5a–c) compared to the previous case. The higher surface roughness and developed interfacial area ratio in the case of SH100 are induced precisely by the sizes of the organized formations, but also by the nanoscale morphological details found on the hilltops. 

SN-type supramolecular azo-polyimides have a greater auto-organization capacity compared to the ones containing azobenzene with CH_3_ group. This can be observed in the AFM morphological analysis from Figure 6 and Figure 7. SRGs are no longer observed with a spiral appearance but rather with a compact appearance, slightly interrupted when the amount of azo-chromophore is lower, but very well defined when the molar ratio between the polyamidic acid and azomonomer is increased. In addition, unorganized material between the modulations is no longer visible in the amplitude images, confirming once again that the use of AzoCN gives better results. According to the profile images and the performed statistics, the SRGs keep almost the same width, and the height increases by more than 100 nm for SN100, compared to SN50. This causes Sq and Sdr to increase significantly, together with the increase in the complexity of the surface morphology. 

The close to 0 values obtained for the Stdi parameter (Table 3) are an indicator that the azo-polyimides with supramolecular organization develop surfaces with anisotropic morphology after phase mask UV-laser irradiation, comparing to the isotropic morphology developed by S0 in the same exposer conditions. The best results were evidenced once again for SN100, with the Stdi reaching 0.162. The orderly aspect of the surface formations was highlighted by means of the autocorrelation images (Figure 4d, Figure 5d, Figure 6d and Figure 7d) and those of the 2D fast Fourier transform (Figure 4e, Figure 5e, Figure 6e and Figure 7e), as well as the corresponding profiles (Figure 4d’, Figure 5d’, Figure 6d’, Figure 7d’, Figure 4e’, Figure 5e’, Figure 6e’ and Figure 7e’). The autocorrelation and 2D FFT counts are visibly higher for the sample SN100 with a higher structuring capacity. Derived from these measurements, the very low values calculated for the texture aspect ratio indicate for all the samples a high uniformity of the orientation. 

### 2.4. Molecular Modeling

The molecular modeling was applied to explain the nanoscale comportment of the polymers under investigation throughout the course of the morphological organization of surface relief gratings as a consequence of phase mask UV laser irradiation. Since the experimental AFM determinations were performed on laser-irradiated samples, the behavior of pristine polyimide and polyimide-based supramolecular systems was pursued through molecular modeling. The systems containing both types of azo-chromophores were studied in different *trans* and *cis* configurations. The subject of the research were 13 types of amorphous cells constructed as follows: (1) pristine polymer S0, (2) polymeric systems with azo groups in *trans* configuration (SH50_1, SH100_1, SN50_1 and SN100_1), (3) polymeric systems with azo-chromophores, of which 50% are in *cis* configuration (SH50_2, SH100_2, SN50_2 and SN100_2), and (4) polymeric systems with the azo-dyes in *cis* configuration (SH50_3, SH100_3, SN50_3 and SN100_3). Table 4 lists the percentages of the groups in the *cis* configuration for each polymer system at the end of the NVT (constant atom number, volume, and temperature) molecular dynamics. It was found that all systems were well balanced, with the density reaching a plateau value.

**Figure 8:**
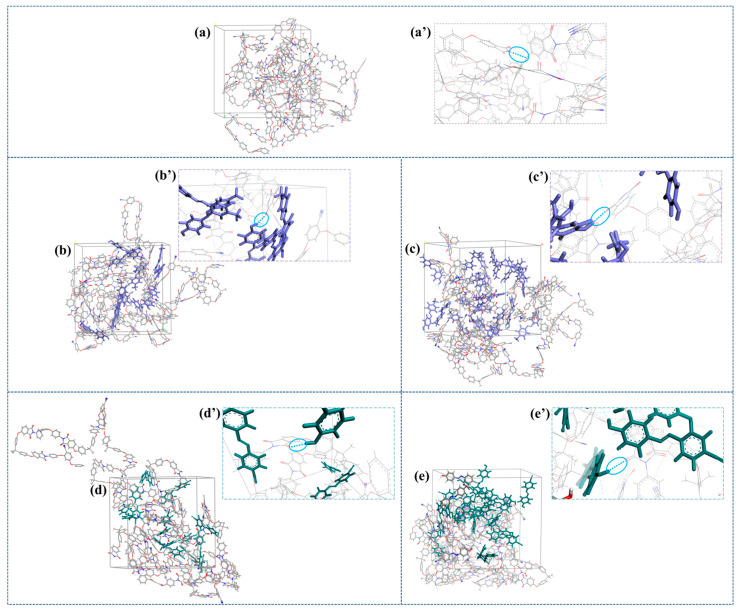
Amorphous unit cells of (**a**) S0, (**b**) SH50_1, (**c**) SH100_1, (**d**) SN50_1 and (**e**) SN100_1. Atom representation: carbon-grey, hydrogen-white, oxygen-red, azo groups in SH-purple, azo groups in SN-dark green. The hydrogen bond is highlighted for each case: (**a’**) S0, (**b’**) SH50_1, (**c’**) SH100_1, (**d’**) SN50_1 and (**e’**) SN100_1.

When the geometry of the systems is favorable for the formation of hydrogen bonds, they were highlighted both in the amorphous polyimide cells and in those with azochromophores (Figure 8). The intramolecular hydrogen bonds formed between the oxygen from the main chain and the hydrogen linked to the nitrogen atom at the ends of the chain contribute to a globular conformation of the polymer. In the SH and SN type systems, a multitude of intermolecular hydrogen bonds were formed between the protons of azochromophores and oxygen of the imide groups from the polyimides backbone.

**Figure 9:**
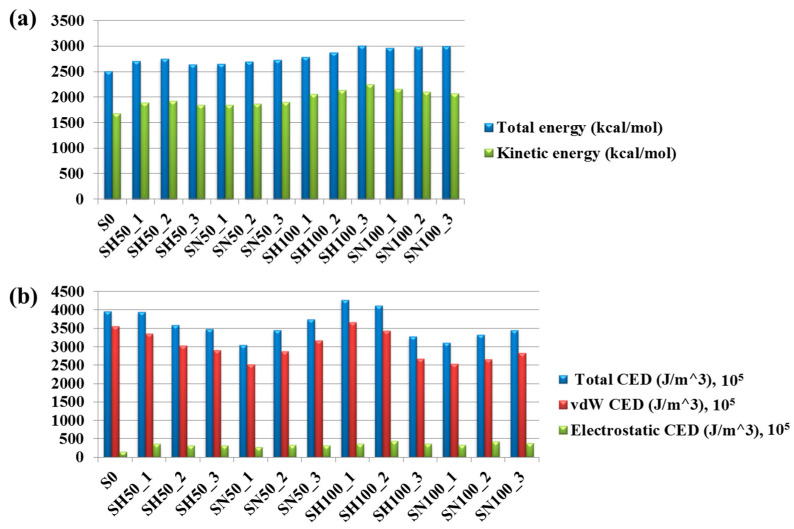
(**a**) Illustration of changes in total and kinetic energy of the simulated systems; (**b**) Total, van der Waals and electrostatic CED of the polyimides systems.

For the energy analysis of the systems, the total and kinetic energies were evaluated. The system’s energetic parameters at 298 K are illustrated in Figure 9a. It can be seen that the total energy of the SH100 and SN100 supramolecular systems is higher than that of the corresponding SH50 and SN50 supramolecular systems, respectively. This fact is expected because the number of atoms from the system increases and, at the same time, there is a higher probability of intermolecular H-bond formation when the polyamidic acid and azomonomer molar ratio is 1:1. Comparing the kinetic energy values, it was found that SH100 and SN100 have higher values than the homologous systems SH50 and SN50, respectively. SH 50 systems do not have a linear behavior in terms of the evolution of kinetic energy by passing from one configuration to another (1–3).

The cohesive energy density (CED) is an important bulk material property for the polymers. It is a quantitative measure of the intermolecular interaction and compatibility between the molecules. In Figure 9b, the electrostatic and van der Waals cohesive energy density of the simulated systems at 298 K are plotted. In all cases, the higher attractive van der Waals CED is observed, with values very close to those of the total cohesive energy density. Since the electrostatic CED has very low values for all polymer systems, it means that the biggest contribution to intermolecular interactions is made by the van der Waals forces. In the case of the samples with methyl-type azochromophores, the CED values decrease slightly with the increase in the content of *cis* groups. The SN type systems have an opposite behavior: the CED values have a slight increase, with the increase in the content of the groups in the *cis* conformation. Moreover, SH systems of type 1 and 2 have higher cohesive energy density than SN systems 1 and 2. The higher the cohesive energy density, the stronger the interaction between the molecules is. Experimentally, it was found that the SN systems showcase the most pronounced surface relief gratings than the SH systems, especially when the amount of azo-chromophore increases.

To analyze the behavior of the system from a dynamic point of view, the Mean Square Displacement (MSD) parameter was used. This parameter provides information about the mobility of the particles from the system. The movement is described by the relative position of a particle or a group of particles with respect to a reference position, in a time interval [36]. The MSD values of the polymer chains from the simulated systems are listed in Table 5. It can be observed that MSD values for the systems SH and SN are lower than S0 in the initial state, meaning 0% content of *cis* azochromophores. Therefore, the presence of small molecular compounds worsens the mobility of the polyimide chains. Things change substantially when the polymer systems have a maximum content of azo groups in the *cis* configuration, and, among them, the SN_3 systems stand out for their much higher values. A possible explanation would be the creation of a free volume and the increase of the interchain distances through the contortion of the azochromophore molecules. 

**Figure 10:**
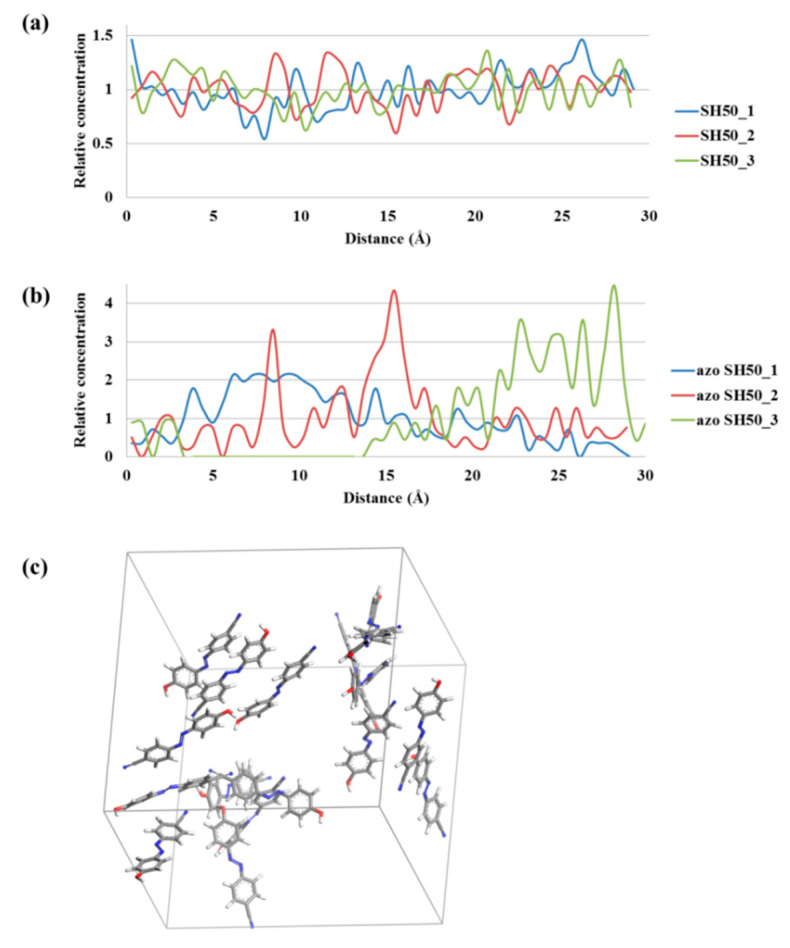
Concentration profiles of (**a**) polymer chain SH 50 and (**b**) azo groups along the x axis. Snapshot of azo groups from SH 100_1 (**c**). For a better visualization, the atoms of the polymer chains and some azo groups have been removed.

The concentration profiles of the polymer chains show only small differences regarding the amplitude of the fluctuation of the concentration gradients around the median value 1 (Figure 10a). The atoms’ distribution along the planes (100), (010), and (001) was inhomogeneous, indicating a non-uniform composition at the atomic level and consequently amorphous structures. On the other hand, azo groups have very large fluctuations in concentration both within the same system and from one system to another (Figure 10b). In addition, an association trend was observed. If the geometry allows it, local alignments of the azochromophore groups also take place, as is the case with the SH 100_1 and SN_1 type systems (as exemplified in Figure 10c). This can explain the formation of higher modulations via UV-laser irradiation in the mentioned situations. It seems that the supramolecular reorganization process is enhanced by this phenomenon, since it is determined by azo-groups dipole orientation. 

## 3. Materials and Methods 

### 3.1. Materials

The monomers used in this study, 4,4′-isopropylidene-diphenoxy-bis(phthalic anhydride) (6HDA), were purchased from Sigma-Aldrich, St. Louis, MO, USA, and 2,4-Bis(p-aminophenoxy)benzonitrile (o-CN) was synthesized in our laboratory by a previously reported method [47,48,49]. N,N-Dimethylacetamide (HPLC grade), *p*-toluidine (97%), *p*-aminobenzonitrile, phenol (99%), and NaNO2 (99.9%) were purchased from Merck KGaA, Darmstadt, Germany. All reagents and solvents were used as received. 4-[(4-Methylphenyl)diazenyl]phenol (AzoCH3) and 4-[(4-cyanophenyl)diazenyl]phenol (AzoCN) was synthesized in our laboratory by a previously reported method [50,51]. M.p. were 152–154 °C and 200–202 °C, respectively.

### 3.2. Synthesis of Polyamidic Acid (PAA)

The polyamidic acid (PAA) has been synthesized starting from the 2,4-bis(p-aminophenoxy)benzonitrile (o-CN) and 4,4′-isopropylidene-diphenoxy-bis(phthalic anhydride) (6HDA). This step of the polycondensation reaction was performed with equimolar amounts of o-CN and 6HDA in DMAc at a total concentration of 10–15%, at room temperature, and under an inert atmosphere for 4 h. Polyamidic acid was used to the obtaining of supramolecular systems and at this stage was not isolated.

### 3.3. Synthesis of Polyimide-Based Supramolecular Systems 

**Scheme 1:**
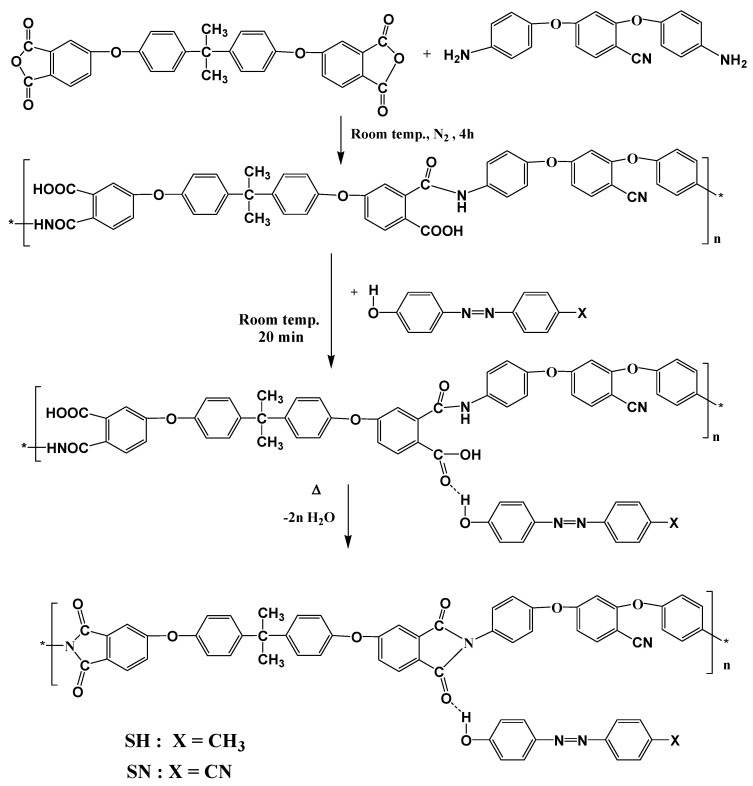
The steeping structural evolution of the reaction that lead to the polyimide-based supramolecular systems. The * signs indicate that the structure it’s repeating in both sides n times.

**Figure A6:**
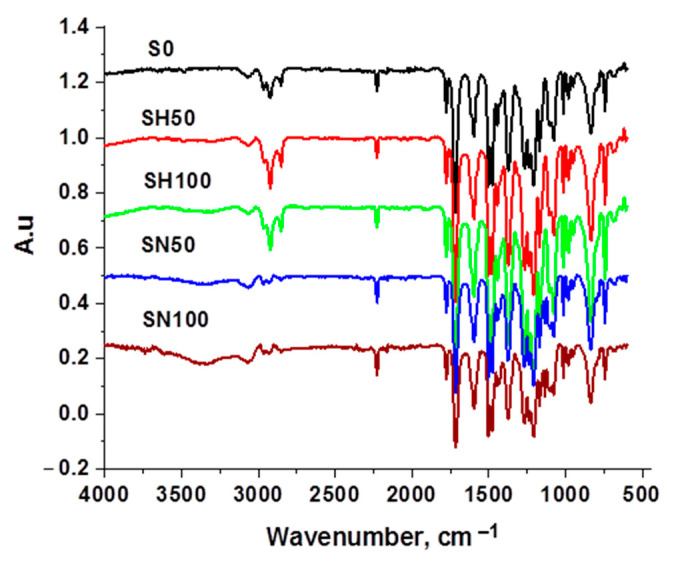
FTIR spectra of pristine polyimide film S0 and supramolecular azo-polyimide films SH50, SH100, SN50 and SN100.

**Figure A7:**
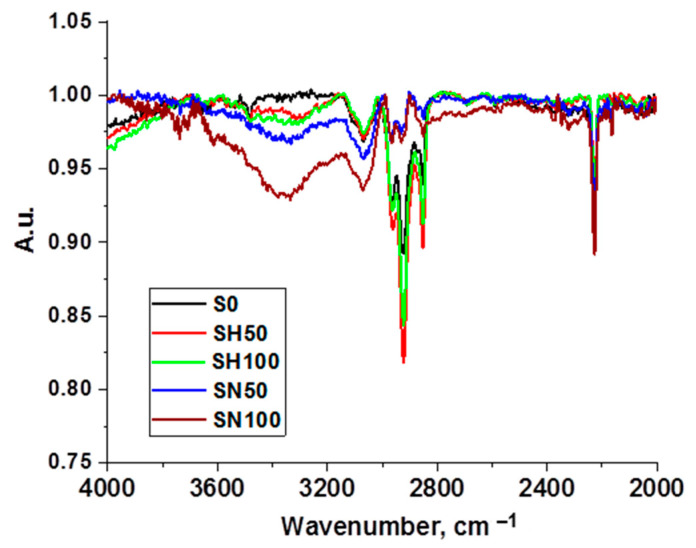
FTIR spectra of pristine polyimide film (S0) and supramolecular azo-polyimide films SH50, SH100, SN50 and SN100) in the range of 4000–2000 cm^−1^.

**Figure A8:**
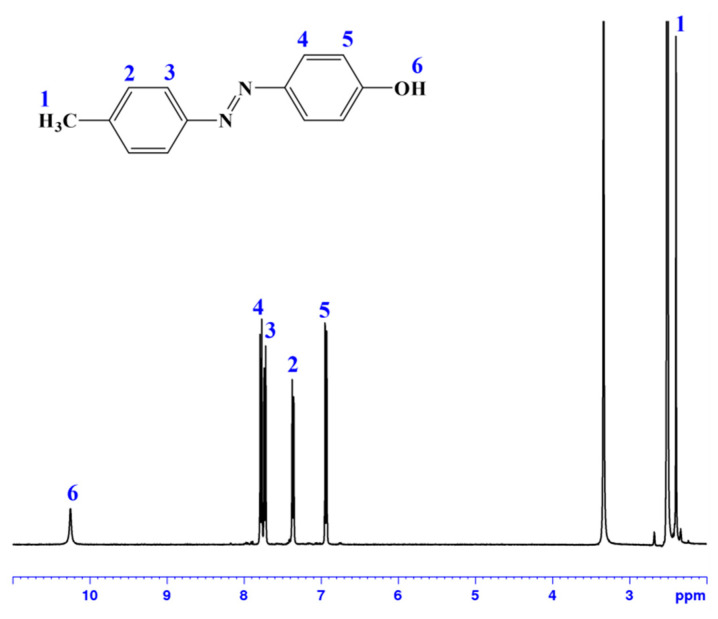
The ^1^H-NMR spectrum of the AzoCH_3_.

**Figure A9:**
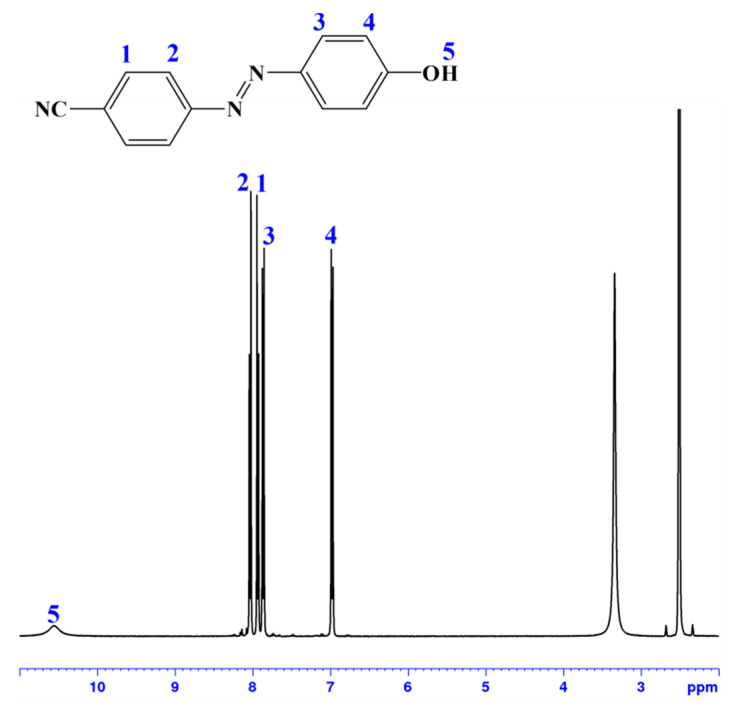
The ^1^H-NMR spectrum of the AzoCN.

**Figure A10:**
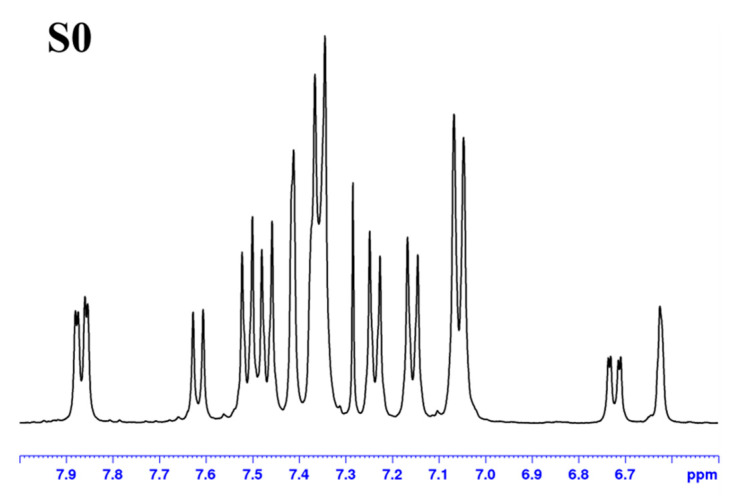
The ^1^H-NMR spectrum of the S0.

**Figure A11:**
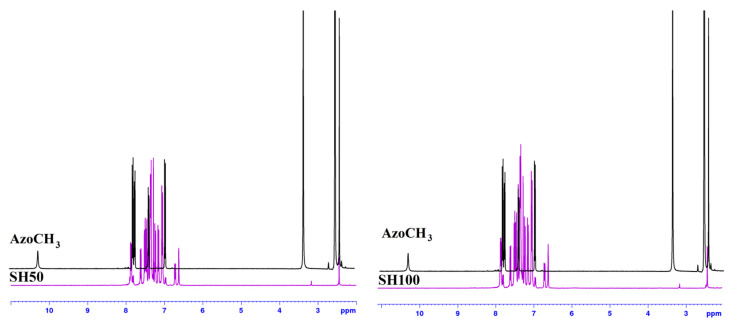
The ^1^H-NMR spectra of the SH50 and SH100.

**Figure A12:**
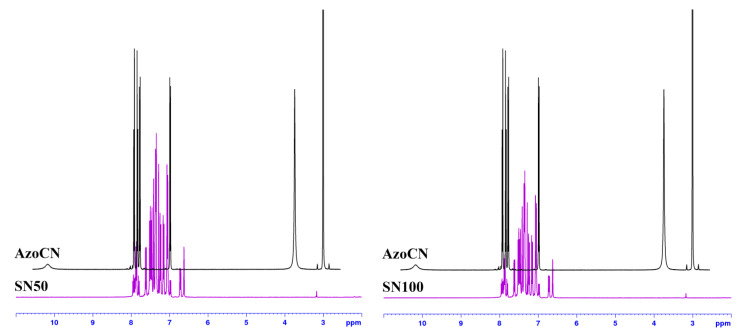
The ^1^H-NMR spectra of the SN50 and SN100.

Polyimide-based supramolecular systems have been realized by dissolving 4-[(4-methylphenyl)diazenyl]phenol (AzoCH3) or 4-[(4-cyanophenyl)diazenyl]phenol (AzoCN) with the aromatic polyamidic acid solution. The molar ratio between polyamidic acid and azomonomer was 1:0.5 (SH50, SN50) and 1:1 (SH100, SN100). The used quantities are presented in Table 6 and the steeping structural evolution of the reaction is shown in Scheme 1. Flexible films were obtained by casting guest-host PAA solution on a glass plate, followed by thermal treatment to realize the imide structure. The thermal treatment starts at 50 °C for 4 h, followed by slowly increasing the temperature at 100 °C, 125 °C, 150 °C, 175 °C, and 200 °C, maintaining it at each step for 1 h. The pristine polyimide film based on o-CN and 6HDA (S0) has been obtained by the same thermal treatment of polyamidic acid without adding azo monomers. The obtained films with thicknesses of 30–40 microns were peeled from the glass plate and used for characterization. The structural characterization of all synthesized compounds was performed by means of Fourier-transform infrared spectroscopy (see Appendix C, Figure A6 and Figure A7) and proton nuclear magnetic resonance (see Appendix D, Figure A8, Figure A9, Figure A10, Figure A11 and Figure A12). 

### 3.4. Methods

The Fourier-transform infrared spectroscopy (FT-IR) was performed on film samples with a FT-IR Bruker Vertex 70 Spectrophotometer in ATR mode.

The proton nuclear magnetic resonance (^1^H-NMR) spectra were recorded on a Bruker Avance III 400 instrument, equipped with a 5 mm multinuclear inverse detection probe, and operating at 400.1 MHz. The chemical shifts were reported in δ units (ppm) relative to the residual peak of the solvent. 

The illuminance properties of the pristine and supramolecular azopolyimide films were determined on a CRI Illuminance Meter CL-70F (Konica Minolta, INC., Tokyo, Japan), a portable spectrometer supplied with a complementary metal-oxide-semiconductor (CMOS) linear image sensor that can perform measurements from 380 nm to 780 nm. 

The thermal stability of the polyimides was studied by thermogravimetric analysis, which was performed on a Thermogravimetric Analyzer, Discovery TGA 5500 (TA Instruments, New Castle, DE, USA) under a nitrogen flow, at a heating speed of 10 °C/min, from 50 to 700 °C. The initial mass of the samples was 5 mg.

The UV-Vis absorption spectra were carried out on a Shimadzu UV-1280 UV-Vis spectrophotometer (Shimadzu Scientific Instruments, Kyoto, Japan).

The UV laser patterning of the pristine polyimide and the supramolecular azo-polyimide films was realized by means of a Brilliant B pulsed Nd:YAG laser (Quantel, Les Ulis Cedex, France), working at a wavelength of 355 nm. A phase mask with 1000 grooves per mm (Edmund Scientific Co., Barrington, NJ, USA) was placed prior to the samples. Quartz plates with a thickness of 1 mm were placed between the extended laser beam (from 5 mm to 15 mm) and the phase mask, the phase mask and the samples, and after the samples. The grating region was equally illuminated since the laser was horizontally polarized. The determined value of the incident fluency after the beam expander was 30 mJ/cm^2^. The duration of one pulse was 6 ns. The number of pulses was 100. Each pulse was repeated at a rate of 10 Hz. 

The surface topography of the UV laser irradiated films was investigated using a NTEGRA Scanning Probe Microscope (NT-MDT, Zelenograd, Moscow, Russia), in semicontact error mode, with a NSG10 cantilever (NT-MDT, Zelenograd, Moscow, Russia). The resonance frequency of the cantilever was 300 kHz. The dimensions of the cantilever were: length L = 95 ± 5 μm, width W = 30 ± 3 μm, thickness T = 2 ± 0.5 μm). The measurements were performed with a vertical deflection setpoint of 10 nA and a scan frequency of 0.8 Hz, in atmospheric conditions, at 23 °C room temperature. Nova software 1.0.26.1443 from NT-DMT was employed to control the acquisition process and to analyze the recorded data. Image Analysis 3.5.0.19892 software was used to calculate the texture parameters on the selected imaged scan size of 10 × 10 μm^2^.

The building and molecular dynamics (MD) simulations of polymeric models were conducted in the Materials Studio [52]. In order to simulate the behavior at the nanoscale level, first the polymer chains consisting of ten units were built, with the units being previously minimized at the quantum level within the DMol3 module using the Perdew-Wang PWC functional. Through the option of connecting the structural units between them, chains with coil and linear conformation were obtained. The chains were subjected to an energy minimization process (Forcite—0.0001 kcal/mol convergence energy). A pcff force field was the force field used in all mechanics and molecular dynamics calculations. After the geometric optimization, two chains with different conformations were introduced into the cubic simulation cells with periodic boundary conditions, starting from an initial density of 0.1 g/cm^3^. Thirteen amorphous cells containing only polymer chains or polymer chains with azo-chromophore groups were constructed, the latter category being in different *cis* and *trans* configurations. In order to minimize the total energy, the bulk systems were subjected to a geometry optimization procedure. To overcome the energetic barriers and to find the energetically favorable minima much faster, molecular dynamics were conducted in the canonical ensemble (NVT). Three heating and cooling repeating processes between 300 and 900 K, summing 500 ps, were performed. After another NVT dynamics at 298 K for 500 ps, the systems were well balanced. Afterwards, in order to achieve the equilibrium, MD simulations in the isothermal-isobaric ensemble (NPT) were applied on the polymer systems for 1000 ps [37,53]. A total of 1 atm pressure and 298 K were considered. To control the temperature and pressure during MD simulations, the Nose thermostat and Berendsen barostat have been set. A cutoff of 12.0 Å is used for non-bond interactions.

## 4. Conclusions

New aspects derived from the features of the supramolecular azopolyimides, obtained using two types of azo-monomers, AzoCH_3_ or AzoCN that were mixed with a polyamidic acid (PAA), were explored. The obtained supramolecular systems processed in the form of flexible films resistant to multiple bends and twists were also thermostable. Regarding the visual aspect, the colorimetry study indicates that the peak wavelength, color temperature, illuminance and transmittance vary depending on the nature and amount of azo-chromophore used in the reaction. The supramolecular materials with AzoCH_3_ have a shade of yellow, while supramolecular materials with AzoCN are orange in hue. The coloring was more intense as the azo-dye concentration increased. SH50 containing the lowest amount of AzoCH_3_ presented the highest illuminance and transmittance, while SN100 containing the highest quantity of AzoCN had the lowest values for the mentioned parameters. 

The supramolecular azo-polyimide containing CN-based azo-chromophore had the best capability to form surface relief gratings on the action of the UV laser, through a phase mask with grooves, and implicitly the greatest anisotropy of the morphology for SN100. In addition, it was found that increasing the amount of azo-chromophore, regardless of its nature, will lead to an increase in the heights of the formed relief modulations.

Different simplified models have been constructed for the theoretical analysis of polymer systems to explain their behavior under UV-laser irradiation. It was found that the systems with a molar ratio of 1:1 between polyamidic acid and azomonomer have the highest kinetic energy values. These are the systems that give the highest relief heights through laser irradiation. From the data on cohesive energy density, it was noted that the van der Waals forces have a greater contribution in intermolecular interactions, in favor of electrostatic interactions. From the MSD analysis, it was observed that the polyimide systems that have the maximum content of azo groups in the *cis* configuration have better mobility of the polymer chains compared to the other systems. Probably bulky *cis* azo groups create the free spaces that give a freedom of movement of the polymer chains. On the other hand, the grouping and even the association of the azo groups in the *trans* configuration, seen from the concentration profile, prevents the movement of the polymer chains—a fact certified by the lower values of the Mean Square Displacement, even compared to the pristine ones.

The studied flexible supramolecular azo-polyimide films were morphologically designed by inducing on their surface SRGs to be tested as substrates for applications in flexible polymer memory devices. The improvement of the surfaces by laser-generated micro patterns on a generous area will be used to enhance the data storage ability, boosting the memory cell response.

## Figures and Tables

**Table 1:** The values of the parameters extracted from the illuminance measurements without sample, and for the samples S0, SH50, SH100, SN50 and SN100.

Sample	λ_pk_ (nm)	Tcp (K)	E_v_ (lx)	T (%)
three normal light LEDs	486	6643	465	-
S0	452	5713	388	83
SH50	579	3485	278	59
SH100	582	3338	246	52
SN50	585	2888	224	48
SN100	590	2570	170	36

λ_pk_: peak wavelength; Tcp: color temperature; E_v_: illuminance; T: transmittance.

**Table 2:** Thermal characterization of pristine polyimide film S0 and supramolecular azo-polyimide films SH100 and SN100.

Sample	T_onset_, °C	T_5_, °C	T_max_, °C
S0	512	-	546
SH100	228	308	537
SN100	247	308	532

T_onset_: thermal degradation onset temperature; T_5_: initial decomposition temperature; T_max_: temperature of the maximum decomposition rate.

**Table 3:** The dimensions of the occurred structures and the AFM surface texture parameters calculated on the selected 10 × 10 μm^2^ images obtained for phase mark UV laser irradiated pristine polyimide (S0) and supramolecular azo-polyimide films (SH50, SH100, SN50, SN100).

Sample	Type of Structuration	H (nm)	W (nm)	Sq (nm)	Sdr (%)	Stdi	Str
S0	Cone-like structures	45 ± 15	667 ± 10	6.8	0.366	0.556	0.483
SH50	Surface relief gratings	53 ± 2	592 ± 55	11.7	0.672	0.286	0.074
SH100	Surface relief gratings	99 ± 6	532 ± 27	18.7	2.041	0.179	0.052
SN50	Surface relief gratings	88 ± 3	670 ± 5	22.1	1.752	0.189	0.078
SN100	Surface relief gratings	198 ± 11	672 ± 7	52.9	8.866	0.162	0.076

H: height; W: width; Sq: root mean square roughness; Sdr: surface area ratio, Stdi: texture direction index; Str: texture aspect ratio.

**Table 4:** The percentage of azo groups in the *cis* configuration and the density of the simulated systems.

System	S0	SH50_1	SH50_2	SH50_3	SN50_1	SN50_2	SN50_3
**% *cis***	-	0	30	80	0	20	80
**Density (g/cm^3^)**	1.235	1.186	1.207	1.218	1.238	1.229	1.232
**System**		**SH100_1**	**SH100_2**	**SH100_3**	**SN100_1**	**SN100_2**	**SN100_3**
**% *cis***		0	30	80	40	0	75
**Density (g/cm^3^)**		1.201	1.219	1.205	1.223	1.215	1.227

**Table 5:** MSD values of polyimide chains in the amorphous cells.

System	S0	SH50_1	SH50_2	SH50_3	SN50_1	SN50_2	SN50_3
**MSD**	1.71	1.03	1.46	1.84	0.73	1.27	2.94
**System**		SH100_1	SH100_2	SH100_3	SN100_1	SN100_2	SN100_3
**MSD**		1.13	0.91	1.25	0.80	1.21	3.11

**Table 6:** The quantities of polyamidc acid and azo monomer used in the polyimide-based supramolecular systems.

Sample Code	Polyamidic Acid mol (g)	Azo Monomer AzoCH_3_ mol (g)	Azo Monomer AzoCN mol (g)
SH50	0.9768 × 10^−4^ (0.0818)	0.4889 × 10^−4^ (0.010354)	-
SH100	0.9768 × 10^−4^ (0.0818)	0.9768 × 10^−4^ (0.020708)	-
SN50	0.9768 × 10^−4^ (0.0818)	-	0.4889 × 10^−4^ (0.01089)
SN100	0.9768 × 10^−4^ (0.0818)	-	0.9768 × 10^−4^ (0.02178)

## Data Availability

Not applicable.

## Appendix B

The UV-Vis spectra of the synthesized compounds used in this paper are presented in the Figure A2, Figure A3, Figure A4 and Figure A5.

In the case of dyes with the phenol structure (Figure A3), the presence of the methyl substituent accelerated the *cis*-*trans* isomerization process contrary to compounds with hydroxyalkoxy-substituents, for which the fastest isomerization was observed for derivatives bearing nitrile groups [54]. Based on the results obtained by using the AzoCH_3_ chromophore, in comparison to AzoCN, it can be seen in the UV-Vis spectra (Figure A4 and Figure A5) the influence of the azochromophore on the behavior of the supramolecular azocompounds SH and SN. Taking into consideration that the quantity of the azochromophore is low compared to the quantity of the polymer matrix, the behavior of the supramolecular azopolyimide compounds to the isomerization process was good enough. A slightly powerful *trans*-*cis* isomerization was observed for the azocompounds SH, as can be seen in the UV-Vis spectra (Figure A4).

## Appendix C

The FTIR spectra of the synthesized compounds are presented in Figure A6. The FTIR spectrum of pristine polyimide film (S0) shows the absorption characteristic bands for carbonyl group of the imide ring at about 1775 cm^−1^ (asymmetrical C=O imide stretching), 1720 cm^−1^ (symmetrical C=O imide stretching), and the characteristic band for C–N vibration at 1370 cm^−1^ and 735 cm^−1^ prove the formation of imide rings. Other groups were identified as follows: aromatic –C–H linkages due to the absorption bands at 3070–3065 cm^−1^, the characteristic absorption of the CN group at 2230 cm^−1^, the CH_3_ groups from isopropylidene units at 2968–2853 cm^−1^, and aromatic ether due to the absorption bands at 1208–1134 cm^−1^ (Figure A6).

The FTIR spectra of supramolecular azo-polyimide films (SH50, SH100, SN50 and SN100) present all the characteristic absorption bands of the pristine polyimide film S0. With the introduction of azochromophore, some specific characteristics of absorption bands, due to the formation of the H-bonds between carbonyl group in imide rings and hydrogen donor of hydroxylic unit of chromophore, can be seen in the FTIR spectra of the azo-compounds (Figure A7). Thus, the slightly more intense absorption bands in the IR spectrum of the supramolecular azo-polyimide films in the range of 3680–3250 cm^−1^ can be observed, which is characteristic of H-bonds. This domain includes absorption bands of the valence vibrations of free or bounded aromatic hydroxyl groups, hydrogen bonds between the carbonyl in imide rings, and OH group from chromophore [15]. The broad absorption band with a maximum of around 3500 cm^−1^ was assigned to the intermolecular H-bonded OH group of the supramolecular azo-polyimide films (Figure A7).

The existence of the hydrogen bonds in the supramolecular azo-polyimide films (SH50, SH100, SN50 and SN100) is shown more clearly in the range of 4000–2000 cm^−1^ of the FTIR spectra. By introducing the AzoCN chromophore, it was possible to indicate a slightly greater intensity in the broad band around 3500 cm^−1^, proving the results obtained by molecular simulations (Figure A7).

## Appendix D

The chemical structures of the synthesized compounds were proved by ^1^H-NMR characterization. Thus, the data regarding the structure of the monomers and polymers are shown in Figure A8, Figure A9 and Figure A10. For the azopolyimides SH50, SH100, SN50, and SN100, the ^1^H-NMR data were represented by comparing the ^1^H-NMR spectrum of the azochromophore with those of the pristine polyimide matrix. It is clearly shown that the azochromophores molecules exist in the final structures of the supramolecular structure of the compounds SH50, SH100, SN50, and SN100.

^1^H-NMR (DMSO-*d*_6_, 400.1 MHz, 25 °C), δ (ppm): 10.25 (1H, s, H6), 7.79–7.76 (2H, d, 8.8 Hz, H4), 7.73–7.71 (2H, d, 8.2 Hz, H3), 7.37–7.35 (2H, d, 8.1 Hz, H2), 6.94–6.92 (2H, d, 8.8 Hz, H5), 2.39 (3H, s, H1).

^1^H-NMR (DMSO-*d*_6_, 400.1 MHz, 25 °C), δ (ppm): 10.55 (1H, s, H5), 8.04–8.02 (2H, d, 8.6 Hz, H2), 7.94–7.92 (2H, d, 8.6 Hz, H1), 7.87–7.85 (2H, d, 8.8 Hz, H2), 6.98–6.96 (2H, d, 8.8 Hz, H5).

^1^H-NMR (CDCl_3_, 400.1 MHz, 25 °C), δ (ppm): 7.79–7.76 (2H, dd, 8.2 Hz, 2.5 Hz), 7.62–7.60 (1H, d, 8.5 Hz), 7.52–7.50 (2H, d, 8.9 Hz), 7.48–7.45 (6H, d, 8.5 Hz), 7.24–7.22 (2H, d, 8.5 Hz), 7.16–7.15 (2H, d, 8.5 Hz), 7.06–7.04 (4H, d, 8.2 Hz), 6.73–6.70 (1H, dd, 8.6 Hz, 2.1 Hz), 6.62 (1H, s).
